# Clinical medicine journals lag behind science journals with regards to “microbiota sequence” data availability

**DOI:** 10.1002/ctm2.656

**Published:** 2021-12-06

**Authors:** Jennifer Pryor, Guy D. Eslick, Nicholas J. Talley, Kerith Duncanson, Simon Keely, Emily C. Hoedt

**Affiliations:** ^1^ School of Biomedical Sciences and Pharmacy College of Health Medicine and Wellbeing University of Newcastle Newcastle Australia; ^2^ NHMRC Centre for Research Excellence in Digestive Health University of Newcastle Newcastle Australia; ^3^ School of Medicine and Public Health College of Health Medicine and Wellbeing University of Newcastle Australia; ^4^ Hunter Medical Research Institute Newcastle Australia

**Keywords:** Keywords data availability statement, microarray, microbiome, sequence data

## 1 BACKGROUND

Microbiota sequencing has received much greater attention over the past 10 years as a result of decreasing sequencing costs and advancing analysis capabilities.^1^, ^2^, ^3^ Despite this, cohort microbiota studies remain beyond the capacity of many researchers with novel hypotheses for a variety of reasons, including access to funding or access to disease cohorts of interest. Despite these limitations, it is possible to collate primary microbiota data from published datasets and conduct secondary analysis to address unique research questions. This approach has been used previously to recover new findings, validate results, and/or increase a studies power.^3^, ^4^, ^5^ The method is most effective when the original publications cite accession numbers for their sequence data deposited in public repositories. Here we report the extent of journals enforcing the inclusion of data availability statements and as a result how we as a Science community are lagging with the public deposit of sequence data hindering scientific progress.

Our research team recently posed a research question and related hypothesis that we believed had the potential to be answered from secondary analysis of published data from studies on the gut microbiota of functional gastrointestinal disorders. However, of the 24 studies identified as having related data, only five had published accession numbers for the associated datasets. Following attempts to contact the remaining 19 corresponding authors, one study author responded with the accession number for the publication date. The remaining corresponding authors have not responded after 5 months despite the fact that six out of 16 of these publishing journals have data availability requirements for published studies. This led us to question how stringently and effectively data sharing requirements are enforced in the field of microbiome research.

## 2 COMMENTARY

To investigate the exact relationship of strict inclusion data availability statements, journal impact factors and journal quartile information, we generated a spreadsheet of journal titles, and their reported impact factors and quartiles from 2020, from categories likely to publish research related to the human microbiota, using InCites Journal Citation Reports (https://jcr.clarivate.com). From this extensive list of journals (*n* = 4123), we selected 150 titles for further analysis. The selected journals were chosen based on microbiota research was within the scope of the journal. Where possible we selected a minimum of two journals for each integer impact factor to ensure that the final selection was representative of the spectrum of impact factors. The final selection consisted largely of journals with a focus within the fields of microbiology, gastroenterology, clinical medicine and discovery science. Of the resulting 150 journal titles, a further 55 were removed because they had not published research articles reporting microbiota sequencing in the past 5 years (Table S1). Two independent reviewers examined the author guidelines for the remaining 95 articles, to assess if journals required the submission of sequencing, microarray data, and inclusion of a data availability statement for publication. Curated data was graphed and analysed using GraphPad Prism 9. Statistics were conducted with STATA v.15.

**FIGURE 1:**
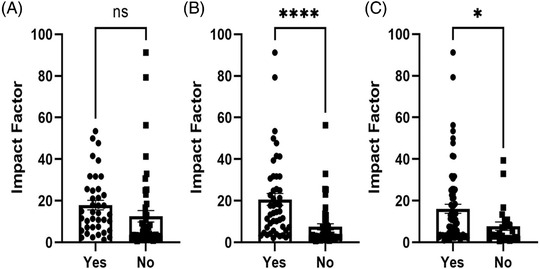
Comparison of journal impact factor with the requirement for submission of (A) sequencing data, 59% of the 95 journals screened did not require submission of sequencing data with the publication, (B) microarray data submission and inclusion of a (C) data sharing statement. Significance level (Unpaired *t*‐test): ns, not significant; *p‐value < 0.05; ****p‐value < 0.0001

Of the selected journals 58% (*n* = 55) were classified as science type journals while the remainder were clinical medicine journals (*n* = 40). Overall, the median impact factor in 2020 was 7.313 (range: 0.747–91.245). The average impact factor of Science journals was 11.87 and for clinical medicine journals, it was 19.95. The median impact by quartile was first quartile, 17.199; second quartile, 4.181; third quartile, 3.267 and fourth quartile, 2.112. Comparing the impact factors by submission of sequencing data revealed a statistically significant difference (17.91 vs. 12.53, *p* = 0.14; Figure 1A) with higher impact factor journals more likely to request sequencing data. The same also occurred for submission of microarray data (20.65 vs. 7.503, *p* = < 0.0001; Figure 1B), and data sharing statements (16.11 vs. 7.797, *p* = 0.0357; Figure 1C).

**FIGURE 2:**
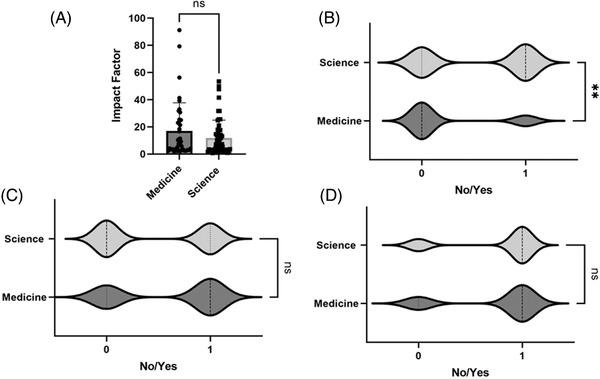
(A) Comparison of impact factors of journals classified as either discovery science (*n* = 55) or clinical medicine type journals (*n* = 40) and whether these journals have requirements for submission of (B) sequencing data, 59% of the 95 journals screened did not require submission of sequencing data with publication (*p* = .0015), (C) microarray data submission and inclusion of (A) and (D) data sharing statement. Significance level (Unpaired *t*‐test): ns, not significant; **p‐value < 0.01

We compared those journals categorized as Science journals compared with Clinical Medicine journals (Figure 2) and found that Science journals were most likely to require sequence data during submission (odds ratio (OR): 6.25, 95% confidence interval (CI): 2.08–16.67, *p* = 0.001; Figure 2B), followed by data sharing statements (OR: 2.38, 95% CI: .90–6.25, p = 0.08; Figure 2D), and finally, microarray data (OR: 2.00, 95% CI: 0.81–5.00, *p* = 0.15; Figure 2C), all adjusted for impact factor.

**FIGURE 3:**
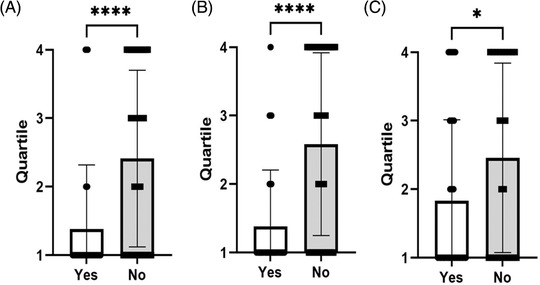
Comparison of journal quartile with a requirement for submission of (A) sequencing data, (B) microarray data and (C) data availability statement. Significance level (Unpaired *t*‐test): *p‐value < 0.05; ****p‐value < 0.0001

We also compared the submission requirements by journal quartile (Figure 3) and found that the journals in the lowest quartile (highest impact factor) were most likely to require microarray data during submission (OR: 0.40, 95% CI: 0.26–0.61, *p* < 0.001; Figure 3B), followed by sequence data (OR: 0.45, 95% CI: 0.29–0.69, *p* < 0.001; Figure 3A), and finally, data sharing statements (OR: 0.68, 95% CI: 0.47–0.98, *p* = 0.04; Figure 3C).

## 3 CONCLUSION

Our findings demonstrate that Discovery Science‐based journals and journals with higher impact factors are more likely to request microbiome data for public access. We propose that access to published data (microbiota sequence or other) should be a standard mandatory requirement for every journal to facilitate reproducibility and the opportunity for novel findings.

## CONFLICT OF INTEREST

Jennifer Pryor, Guy D. Eslick and Emily C. Hoedt declare that they have no conflict of interest. Nicholas J. Talley reports personal fees from Allakos, Aviro Health, from Antara Life Sciences, Arlyx, Bayer, Danone, Planet Innovation, Takeda, Viscera Labs, twoXAR, Viscera Labs, Dr Falk Pharma, Censa, Cadila Pharmaceuticals, Progenity Inc, Sanofi‐aventis, Glutagen, ARENA Pharmaceuticals, IsoThrive, BluMaiden, HVN National Science Challenge, non‐financial support from HVN National Science Challenge, New Zealand, outside the submitted work. Nicholas J. Talley has a patent Biomarkers of IBS licensed (#12735358.9‐1405/2710383 and (#12735358.9‐1405/2710384), a patent Licensing Questionnaires Talley Bowel Disease Questionnaire licensed to Mayo/Talley, a patent Nestec European Patent licensed, and a patent Singapore Provisional Patent NTU Ref: TD/129/17 “Microbiota Modulation Of BDNF Tissue Repair Pathway” issued and copyright, Nepean Dyspepsia Index (NDI) 1998 and Editorial: Medical Journal of Australia (Editor in Chief), Up to Date (Section Editor), Precision and Future Medicine, Sungkyunkwan University School of Medicine, South Korea, Med (Journal of Cell Press). Nicholas J. Talley has participated in the following committees: Australian Medical Council (AMC) Council Member (2016–2019), MBS Review Taskforce (2016–2020), NHMRC Principal Committee, Research Committee (2016–2021), Asia Pacific Association of Medical Journal Editors (APAME) (current) and GESA Board Member (2017–2019). Nicholas J. Talley (Miscellaneous): Avant Foundation (judging of research grants) (2019). Nicholas J. Talley community and patient advocacy groups: Advisory Board, IFFGD (International Foundation for Functional GI Disorders). Nicholas J. Talley acknowledges funding from the National Health and Medical Research Council (NHMRC) for the Centre for Research Excellence in Digestive Health. Nicholas J. Talley holds an NHMRC Investigator grant.

Kerith Duncanson is a company director for the Good Gut Group, which has patented functional bread and grain product concepts (Australian Patent No. 2014262285; New Zealand Patent No. 629207; South Africa Patent No. 2015/07891) for Irritable Bowel Syndrome (IBS) consumers. Good Gut Group had no role in the design of the study; in the collection, analyses, or interpretation of data; in the writing of the manuscript, or in the decision to publish the results.

Simon Keely (Grant/research support): National Health and Medical Research Council (Ideas Grant and Centre for Research Excellence) Viscera Labs (Research contract) and Microba Life Science (Research contract). Consultant/Advisory Boards: Gossamer Bio (Scientific Advisory Board), Anatara Lifescience (Scientific Advisory Board) and Microba Life Science (Consultancy).
